# Benchmarks for urine volume generation and phosphorus mass recovery in commercial and institutional buildings

**DOI:** 10.1016/j.wroa.2024.100227

**Published:** 2024-05-08

**Authors:** Lucas Crane, Ashton Merck, Shwetha Delanthamajalu, Khara Grieger, Anna-Maria Marshall, Treavor H. Boyer

**Affiliations:** a School of Sustainable Engineering and the Built Environment (SSEBE), Arizona State University, PO Box 873005, Tempe, AZ 85287-3005, USA; b Department of Applied Ecology, North Carolina State University, Raleigh NC 27606, USA; c Department of Sociology, University of Illinois Urbana-Champaign, USA; d NSF Science and Technologies for Phosphorus Sustainability (STEPS) Center, USA

**Keywords:** Building occupancy, Resource recovery, Source separation, Urine diversion, Water conservation

## Abstract

Phosphorus (P) is a finite resource and necessary nutrient for agriculture. Urine contains a higher concentration of P than domestic wastewater, which can be recovered by source separation and treatment (hereafter urine diversion). Commercial and institutional (CI) buildings are a logical location for urine diversion since restrooms account for a substantial fraction of water use and wastewater generation. This study estimated the potential for P recovery from human urine and water savings from reduced flushing in CI buildings, and proposed an approach to identify building types and community layouts that are amenable to implementing urine diversion. The results showed that urine diversion is most advantageous in CI buildings with either high daily occupancy counts or times, such as hospitals, schools, office buildings, and airports. Per occupant P recovery benchmarks were estimated to be between 0.04–0.68 g/cap·d. Per building P recovery rates were estimated to be between 0.002–5.1 kg/d, and per building water savings were estimated to be between 3 and 23 % by volume. Recovered P in the form of phosphate fertilizer and potable water savings could accrue profits and cost reductions that could offset the capital costs of new urine diversion systems within 5 y of operation. Finally, urine diversion systems can be implemented at different levels of decentralization based on community layout and organizational structure, which will require socioeconomic and policy acceptance for wider adoption.

## Highlights

• Water savings and recovered P can make urine diversion economically viable.
• Occupancy-based benchmarks can define a building's viability for urine diversion.
• Urine diversion is favorable in hospitals, schools, office buildings, and airports.
• Urine diversion can be scaled spatially depending on a community's layout.

## Graphical abstract

**Figure:**
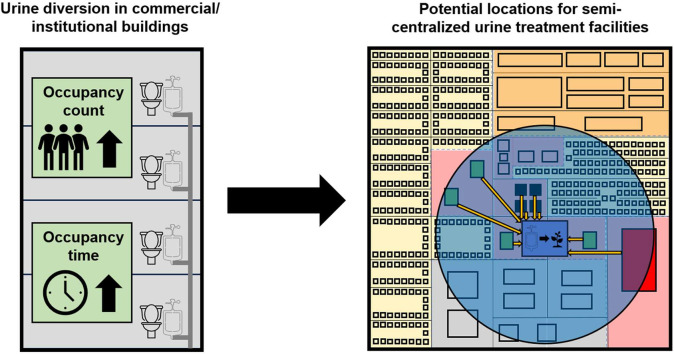

## 1 Introduction

Phosphorus (P) is a key component of commercial fertilizers. However, P is limited in nature, as it is sourced from phosphate rock reserves that are unevenly distributed geographically. Furthermore, P has geopolitical and economic considerations, as over 85 % of global phosphate rock reserves are controlled by five countries with the majority within Morocco and Western Sahara (Garside, 2022). Phosphorus management also involves limiting run-off and loss of P to aquatic ecosystems, where excess P can result in eutrophication that can lead to harmful algal blooms, marine dead zones, and overall degraded ecosystems, along with adverse impacts to drinking water sources (Burkholder and Glibert, 2013; Wurtsbaugh et al., 2019). Given increasing demand for phosphate fertilizers due to global population growth and food security, more sustainable sources of P would advance P-related circular economy initiatives (Neczaj and Grosser, 2018; Robles et al., 2020).

Human urine is a waste stream that generates P at a rate of approximately 1.0 g/cap·d (Kujawa-Roeleveld and Zeeman, 2006; Meinzinger and Oldenburg, 2009; Rose et al., 2015). Urine could be used to augment the production of P fertilizers via source separation and treatment processes to selectively recover P (hereafter referred to as urine diversion). Previous studies have evaluated how urine diversion can affect wastewater systems (Badeti et al., 2021; Hilton et al., 2021; Wilsenach and van Loosdrecht, 2003). These studies showed that implementing urine diversion would improve centralized wastewater treatment operations, as urine contributes 80 % of the total nitrogen and 50 % of the total P in domestic wastewater (Wilsenach and van Loosdrecht, 2003). Previous studies have also evaluated how specific treatment processes can be integrated into urine treatment systems at the point-of-use or after storage and/or transport, showing that different treatment configurations can have environmental benefits and allow for recovery of different P products (Hilton et al., 2021; Kavvada et al., 2017). While previous studies have suggested that implementing urine diversion can benefit wastewater systems and P recovery, minimal literature exists that identifies where urine diversion systems should be located.

Commercial and institutional (CI) buildings may be a suitable location for implementing urine diversion because toilets and urinals account for more of the total water use and wastewater generation compared with residential buildings (DeOreo et al., 2016; Dziegielewski et al., 2000), and implementing urine diversion would have a minimal impact on building occupants (Boyer and Saetta, 2019; Ishii and Boyer, 2016). Additionally, CI buildings often have plumbing designs that are suitable for urine diversion, such as stacked bathrooms that minimize piping needs and maintenance rooms that can house urine storage tanks. For example, previous research studied urine diversion in a multi-story CI building with dedicated urine-only drainage pipes and demonstrated that diverted urine can be processed into different fertilizers (Jagtap and Boyer, 2018, 2020). Another study on a multi-story CI building showed that different occupancy and water usage patterns can affect water quality (Richard et al., 2020). Previous literature has proposed benchmark values for water usage in CI buildings based on occupancy, floor area, and other building metrics (Carvalho et al., 2013; Dziegielewski et al., 2000; Özlem Vurmaz and Boyacioglu, 2018), which allow for estimation of water supply needs for a community. However, no benchmarks were found that predicted wastewater generation for different building types. Wastewater benchmarks for urine generation and P recovery in buildings would be particularly useful since there is an absence of codes and standards for implementing urine diversion systems. Furthermore, urine diversion systems have had issues with precipitation within piping systems due to the urea hydrolysis reaction, which increases pH and creates favorable conditions for phosphate to precipitate (Rosemarin et al., 2012; Udert et al., 2003; Yan et al., 2021). However, dosing of urine diversion systems with acids or bases has been shown to inhibit urea hydrolysis and prevent uncontrolled precipitation within pipes (Randall et al., 2016; Saetta et al., 2019; Saetta and Boyer, 2017), and developing wastewater benchmarks would be useful for creating predictive dosing schemes to ensure continued operation of urine diversion systems.

While nutrient recovery is often given as the main benefit of implementing urine diversion (Randall and Naidoo, 2018), water savings may be an overlooked benefit. For instance, immediate water savings could incentivize replacing flush toilets and urinals with urine-diverting fixtures, which in turn would provide the basis for nutrient recovery. Globally, water availability is a major challenge with half of the world's population expected to experience water scarcity by 2050 (He et al., 2021). Previous literature shows that toilet and urinal flushing contributes >30 % of the total water use in CI buildings (Dziegielewski et al., 2000; US EPA, 2012), and previous studies show that conversion from flush toilets and urinals to low-flush or non-water fixtures can provide major water savings (Boyer and Saetta, 2019; Chipako and Randall, 2019; Kavvada et al., 2017). While these studies have modeled water savings for specific buildings or in individual communities, no studies were found that compared water savings across different CI buildings that implemented urine diversion.

The goal of this research was to provide an improved understanding of where urine diversion should be implemented considering building type and community layout. The research was conducted at two different spatial scales: first at the building-level and then at the community-level. The specific objectives of the research were to develop benchmarks for urine volume generation and P mass recovery potential for different building types, evaluate potential water savings from urine diversion for different building types, determine potential payoff periods of retrofitting wastewater plumbing in buildings for urine diversion, and suggest an approach by which urine diversion can be implemented at the community-scale based on insights from different building types.

## 2 Results and discussion

### 2.1 Benchmarks for urine generation and P recovery for different buildings

To select CI buildings for this study, different types of buildings were screened based on occupancy characteristics, availability of water use benchmarks, and likelihood of having dedicated building staff for managing urine diversion (Section 5.1 and Table S1). As a result, six types of CI buildings were selected for urine diversion benchmarking: hospitals, hotels/motels, office buildings, restaurants, schools/colleges, and airports. These buildings generally had high daily occupancy counts and/or occupancy times, resulting in high potential for urine generation and subsequent P recovery. Furthermore, each of the building types had established water use benchmarks (Dziegielewski et al., 2000; Healthcare Facilities Today, 2013; Özlem Vurmaz and Boyacioglu, 2018). Schools were further divided into primary, secondary, and college/university based on different occupancy and building characteristics.

In this study, benchmarks are defined in units of amount per capita per day, and rates are defined in units of amount per day (Section 5.2). Urine generation benchmarks for different building types are directly related to how long occupants are awake in buildings (Table 1). Buildings with higher occupancy times, e.g., hospitals and office buildings, have greater potential for urine generation, assuming an even distribution of urination events during the time that occupants are awake. In turn, higher urine generation benchmarks correlate with higher P recovery benchmarks (Table 1). In the special case of buildings with 24-h occupants, e.g., hospitals and universities, not only do these buildings have higher occupancy times but they also may have higher P recovery due to first-morning urine having higher P concentrations (Bottin et al., 2016; Perrier et al., 2013; Suh et al., 2020). In addition to the time occupants spend in buildings, P concentration can vary for different occupants based on diet and physiological parameters (McClure et al., 2019; Nadkarni and Uribarri, 2014), or over the course of a day based on hydration (Huang Foen Chung and van Mastrigt, 2009). The influence of variable P concentration on P recovery is explored in more detail below.

**Table 1:** Calculated urine volume generation and corresponding P mass recovery benchmarks.

Building type	Occupant group	Time awake in building per person (h)	Urine generation (L/cap·d)^a^	Phosphorus recovery (g/cap·d)^b^
Hospital	Patients	17.6	1.57	0.68
	Staff	12	1.12	0.46
Hotel/motel	Guests	4	0.37	0.15
	Staff	8	0.75	0.31
Primary school	Occupants	8	0.75	0.31
Secondary school	Occupants	8	0.75	0.31
University/college	Off-campus students	3	0.28	0.12
	Faculty/staff	8	0.75	0.31
	On-campus students	17.6	1.57	0.68
Office building	Occupants	8	0.75	0.31
Restaurant	Customers	1	0.09	0.04
	Staff	4.5	0.42	0.17
Airport	Passengers	1.5	0.14	0.06
	Flight staff	1	0.09	0.04
	Airport staff	8	0.75	0.31

a The typical 24-h urine volume is ∼1.64 L/d (Landry and Boyer, 2016).

b The typical 24-h P mass is ∼0.7 g/d (Prasad and Bhadauria, 2013).

**Fig. 1:**
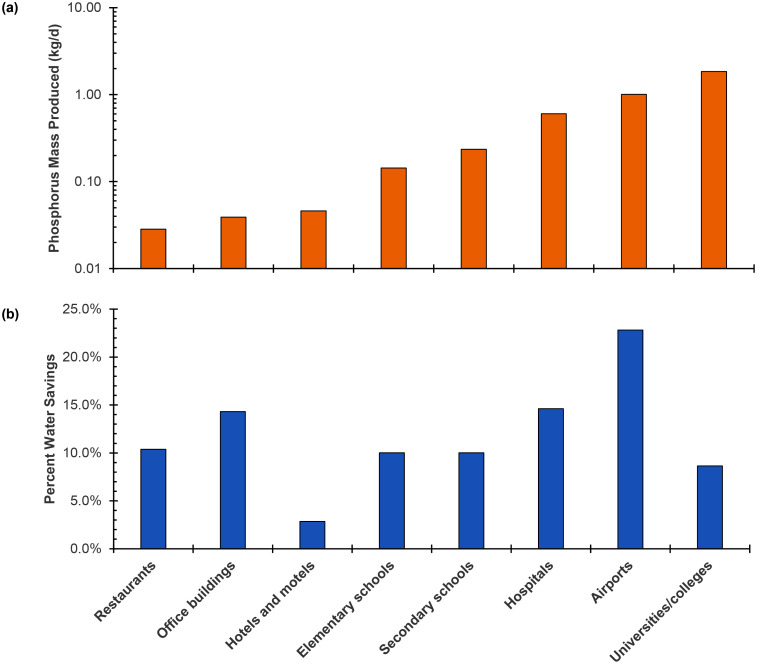
Calculated (a) phosphorus mass recovery rates and (b) water use savings for average occupancy scenario for different CI building types.

Urine volume generation and P mass recovery rates were obtained by multiplying the corresponding benchmarks by low, average, and high occupancy scenarios for different CI buildings (Tables S2–S15). Occupancy scenarios were determined from literature, where the low and high scenarios are extreme occupancy count (Section 5.1). For the average occupancy scenario, the P recovery rate increased in the order restaurants < office buildings < hotels/motels < primary schools < secondary schools < hospitals < airports < universities (Fig. 1(a)), and the P recovery rate varied by a factor of 65 between restaurants and universities. Low and high occupancy scenarios followed similar trends (Figure S1). The urine generation rate followed the same trend for building types as the P recovery rate since P mass is a constant factor of urine volume (Figure S2). The trends in P recovery rate highlight the importance of both occupancy count and occupancy time. For example, airports have relatively low occupancy times (occupants generally spend less than three hours per day in airports) but have significantly higher occupancy counts than other CI building types, resulting in the highest P recovery and urine generation rates. For office buildings, occupants spend eight or more hours per day in buildings (i.e., high occupancy time), but most office buildings have relatively low occupancy counts compared with other CI building types, resulting in lower overall P recovery and urine generation rates. Notably, while this analysis used three potential occupancy scenarios, building managers and urban planners should use occupancy data for specific buildings to determine more accurate P recovery potential.

**Fig. 2:**
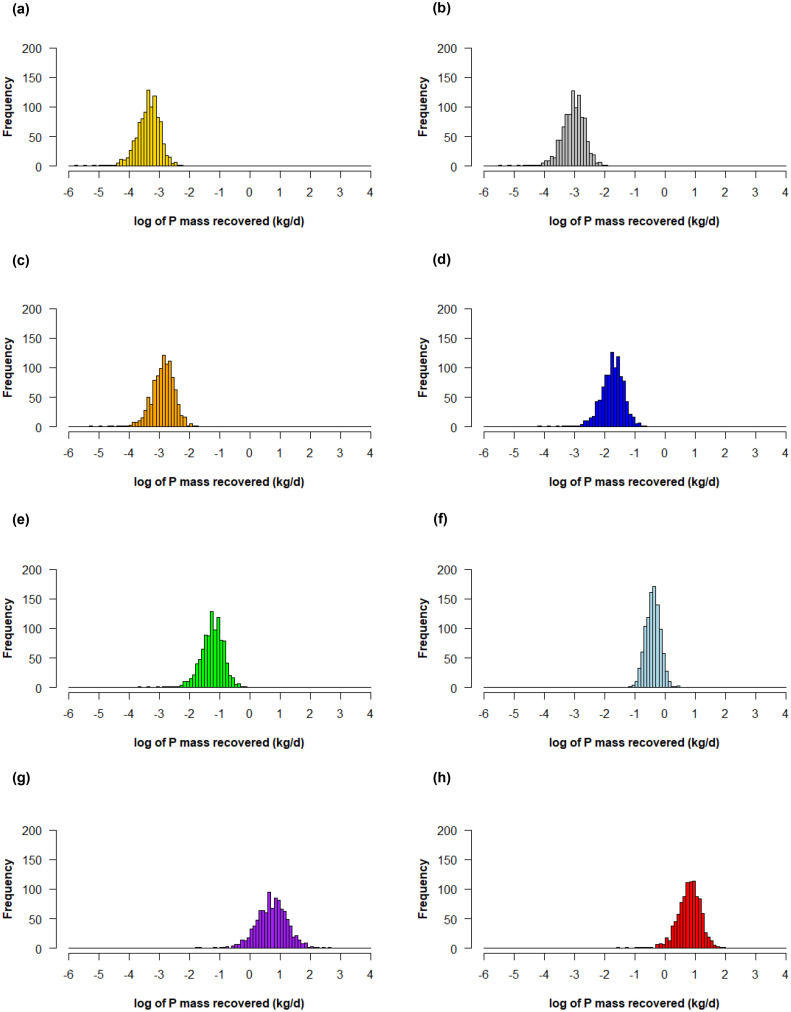
Calculated frequency distribution of P mass recovery rates for average occupancy scenario for (a) restaurants, (b) office buildings, (c) hotels/motels, (d) primary schools, (e) secondary schools, (f) hospitals, (g) airports, and (h) universities/colleges.

Following the single point estimates for P recovery rate based on different occupancy scenarios, distributions of P recovery rate were calculated using daily urine volumes and void volumes for males and females (Figure S3), and varied P concentrations (Figure S4). The P recovery rate distributions were normally distributed when plotted on log scales, with the log of their standard deviations approximately equal to 0.40 kg/d (Fig. 2). The results demonstrate that occupancy count and time remain dominant variables determining urine diversion potential, as the average P recovery rate of each CI building type followed similar trends to single point estimates. However, daily urine volumes, void volumes, and P concentrations can cause variability that may require greater sizing and additional redundancies built into urine diversion systems. It is also important to note that there may be variability in daily urine volumes or urinary P concentrations over a year and over geographic locations due to diurnal or seasonal patterns when considering how hydration can vary for different climatic conditions (Guerrini et al., 1980; Rosinger, 2015; Su et al., 2006) or due to diet or physiological parameters (McClure et al., 2019; Nadkarni and Uribarri, 2014). As discussed earlier, there may also be differences over the course of a day due to daily hydration patterns, especially when considering overnight urine generation by the human body (Huang Foen Chung and van Mastrigt, 2009). In this analysis, literature values for average first-morning urine osmolality and void volumes were used to estimate first-morning urine chemistry for 24-h occupants (Perrier et al., 2013), but it is possible that a wider variability in urine chemistry and void volumes could occur over a day.

### 2.2 Water savings due to implementing urine diversion in buildings

Water savings for different CI buildings were calculated by replacing water-flush urinals and toilets with non-water urinals and urine-diverting toilets (Section 5.3 and Tables S16 through S18). The order of increasing water savings was hotels/motels < universities/colleges < restaurants ≈ primary schools ≈ secondary schools < hospitals ≈ office buildings < airports (Fig. 1(b)). Hotels/motels had the lowest water savings of 3 % v/v from urine diversion because a major portion of their water use is for cooling (Dziegielewski et al., 2000). Office buildings and airports had the highest water savings of 14 % and 23 % v/v, respectively, from urine diversion because most occupants only use water in restrooms. Previous studies show that airports (e.g., due to cooling, irrigation) and hospitals (e.g., due to different hospital activities) have variability in their water-using operations that may reduce water savings from urine diversion to < 20 % and < 10 % v/v, respectively (Batista et al., 2019; Özlem Vurmaz and Boyacioglu, 2018). The results indicate that airports, hospitals, and office buildings have the greatest potential for water conservation from urine diversion (Fig. 1(b)), as toilet and urinal flush water make up a larger portion of their overall water use, which is consistent with water use benchmarking (Dziegielewski et al., 2000). Globally, trends related to improving sustainable building design and operation (e.g., LEED certification (U.S. Green Building Council, 2023)) and increasing water conservation in communities (Moglia et al., 2018; Vickers, 2011) could help facilitate the transition from water-flush toilets/urinals to non-water toilets/urinals.

### 2.3 Cost savings of implementing urine diversion in buildings

An economic analysis compared the capital costs of urine diversion systems to annual profits from recovered P as fertilizer and cost reductions from water savings, and calculated the break-even time where the capital costs equal the sum of the fertilizer profits and water savings for the average occupancy scenario (Section 5.4 and Table S19). (Economic analyses for low and high occupancy scenarios are given in Table S20.) Single-point estimates were used for water cost ($0.0015/gal or $0.0004/L) and P fertilizer cost ($4.1873/kg P), based on ranges of estimates (Ferris, 2014; Quinn, 2022). For the average and high occupancy scenarios, the order of increasing break-even time was hospitals < primary schools ≈ secondary schools < office buildings < universities < hotels < airports < restaurants (Table 2). However, for the low occupancy scenario, the break-even time was the highest for office buildings (Table S20). The results show that capital costs, including urine-diverting fixtures and piping, are paid off by recovered P and potable water savings within 5 y for most CI building types. As airports have a significantly higher occupancy count than other CI buildings, more urine-diverting fixtures are required, resulting in a payoff period of approximately 13 y. Given that urine-diverting fixtures and piping have a lifetime of over 20 y, most recommended CI buildings can accrue cost savings from implementing urine diversion infrastructure. However, small office buildings generally have low occupant-to-appliance ratios, as they have low employee counts but require at least one restroom. Thus, office buildings in the low occupancy scenario had break-even times of greater than the expected lifetime of the urine diversion infrastructure, signifying that such buildings may not be economically feasible for single-building urine diversion implementation.

**Table 2:** Calculated economic analysis of urine diversion systems in CI buildings at average occupancy scenario, including P fertilizer profit (PF) ($/d), water savings (WS) ($/d), wastewater appliance costs (WWC) ($), and break-even time on costs (BE) (y).

Building type	Average scenario			
	PF ($/d)^a^	WS ($/d)^b^	WWC ($)^c^	BE (y)
**Hospitals**	2.5	13	−12000	**2.1**
**Hotels**	0.19	1.0	−2500	**5.6**
**Office buildings**	0.16	0.87	−1300	**3.4**
**Restaurants**	0.12	0.63	−5900	**21**
**Primary schools**	0.60	3.2	−4700	**3.4**
**Secondary schools**	0.98	5.2	−7700	**3.4**
**Universities/colleges**	7.7	41	−75000	**4.2**
**Airports**	4.2	22	−130,000	**13**

a PF calculated from USDA ERS, 2021.

b WS calculated using single-point assumed $0.0015/gal water.

c WWC calculated using an assumed 50 occupants per appliance, assumed number of floors per building type (Table S19), and costs from market averages (Alibaba, 2022; Sloan, n.d.) and personal communications (J. D. Live, personal communication, September 27, 2022).

Notably, cost reductions from water savings are significantly higher than profits from recovered P as fertilizer. This indicates that in near-term scenarios where only urine-diverting fixtures are retrofitted (and urine-only piping and storage tanks are not included), systems will be paid off within 6 y. The effects of water savings and P fertilizer profits on break-even times were further evaluated based on extreme worldwide water prices and P fertilizer costs (Table S21). Global water prices ranged from $0.0002/gal ($0.00005/L) to $0.0251/gal ($0.0066/L) (Tiseo, 2023), while global P fertilizer costs ranged from $1.9272/kg P to $4.7053/kg P (Quinn, 2021, 2023). The results show that water prices had a 54 × greater impact on break-even times than P fertilizer costs, further illustrating the importance of water savings in driving urine diversion implementation. This analysis did not include the capital costs of urine treatment processes or operation/maintenance costs, so the payoff periods discussed here represent a minimum. A variety of P recovery processes exist that have variable capital and operation/maintenance costs, such as phosphate mineral precipitation (Antonini et al., 2011; Ishii and Boyer, 2015; Pradhan et al., 2017; Zhang et al., 2022), membrane separation (Crane et al., 2022), and adsorption (Jagtap and Boyer, 2018).

## 3 Implementation and adoption of urine diversion

### 3.1 Implementation of urine diversion in communities

**Fig. 3:**
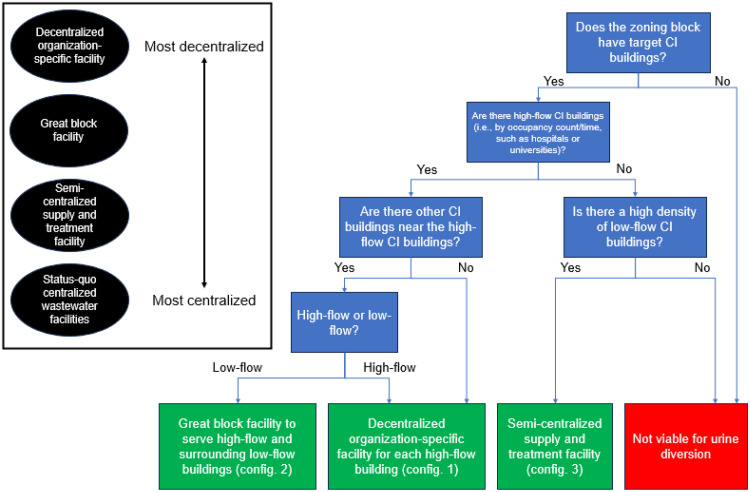
Decision tree for community planners to determine viability of urine diversion in a zoning block.

**Fig. 4:**
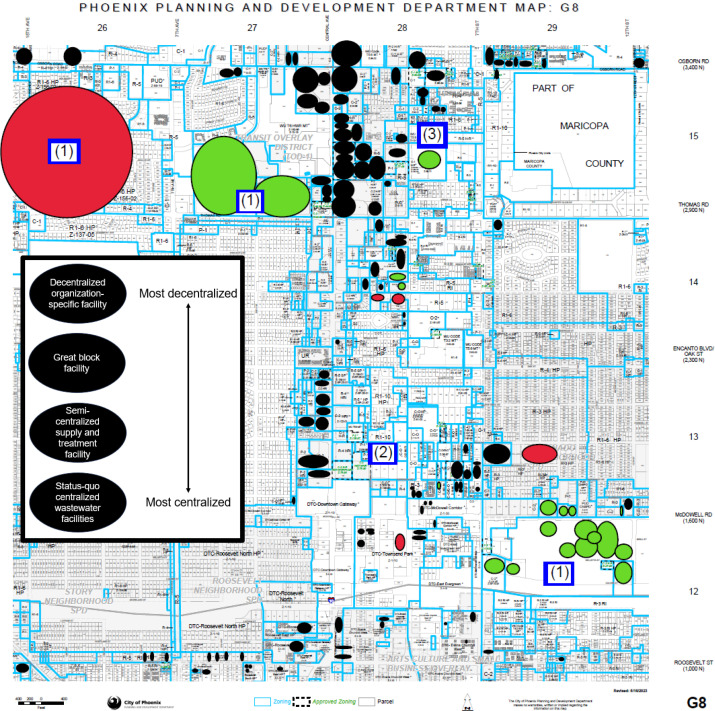
Example of outcomes of proposed approach for implementing urine diversion in a community consisting of schools (red), hospitals (green), and office buildings (black). Urine diversion systems identified with blue box and number indicating level of decentralization (1 = cluster/individual, 2 = great block, 3 = semi-centralized supply and treatment).

Using the urine generation rates determined for CI buildings in this study, P recovery via urine diversion may be implemented in office buildings based on occupancy time as a criterion; airports (if available) based on occupancy count as a criterion; and hospitals, primary and secondary schools, and colleges/universities based on both criteria. Accordingly, a framework was developed (Section 5.5) for implementing urine diversion in a community that considers the types and numbers of CI buildings (hereafter referred to as community layout), including key parameters (Table S22) and a decision tree (Fig. 3). As an example of how the framework may be used by urban planners or other government officials, a zoning block within the Phoenix, Arizona (USA) area (City of Phoenix, 2023) illustrates a possible layout for urine diversion (Fig. 4). The figure displays schools, hospitals, and office buildings within the zoning block and suggests different locations for urine diversion based on community layout and organizational structures, e.g., multiple buildings being managed by a single organization. Urine diversion systems were prioritized in areas with building types that have high urine flows (i.e., schools, hospitals), and in areas with high density of buildings with lower urine flows (i.e., office buildings). Borrowing from terminology in the literature, the level of centralization of water/wastewater treatment infrastructure can be categorized as: fully centralized systems, satellite treatment systems, semi-centralized supply and treatment systems, great block systems, cluster systems, and individual systems (Libralato et al., 2012). While centralized systems have been used for community wastewater treatment for decades, decentralized systems have had growing success for wastewater treatment in individual (Badeti et al., 2021, 2022; Larsen et al., 2009) and great block (Estévez et al., 2023; Kimura et al., 2007) configurations. Accordingly, three configurations of urine diversion were suggested for the zoning block example (Fig. 3, Fig. 4).

In configuration 1, decentralized organization-specific facilities (i.e., cluster or individual) may be implemented that serve a group of buildings under a single organization, e.g., college/university or hospital. This is because of potential high urine flows from an organization and/or different urine contaminants in flows from an organization, e.g., hospital urine may have high concentrations of pharmaceuticals (Blair, 2016; Escher et al., 2011; Langford and Thomas, 2009). In turn, decentralized organization-specific facilities may require different treatment processes at greater scales to manage urine flows. In configuration 2, great block facilities may be implemented in areas that serve a larger group of buildings but are targeted to capture CI buildings with high individual urine diversion potential, e.g., primary or secondary schools. While these targeted buildings may not be large enough to justify a fully decentralized treatment facility, their high urine flows combined with their proximity to other CI buildings (e.g., office buildings) may make a great block system more economically feasible. In configuration 3, semi-centralized supply and treatment facilities may be implemented in areas that serve a larger group of buildings that are densely located, especially office buildings. While individual office buildings do not produce substantial urine flows, areas with a high density of office buildings, e.g., office parks, could collectively produce enough urine to justify a urine diversion system.

Fully centralized or satellite treatment systems are likely infeasible unless a community is mostly made up of CI buildings. Other types of buildings (e.g., residential) do not have high enough occupancy counts, have lower fractions of restroom wastewater generation relative to total wastewater flows, and have low occupant-to-appliance ratios, which each reduce the economic feasibility of implementing urine diversion. While this paper proposes a qualitative approach (as illustrated in Fig. 4 and Section 5.5), a more detailed analysis could be developed using quantitative data from individual buildings or zoning blocks, e.g., building-specific occupancy data, that could inform on locations of urine diversion.

### 3.2 Social considerations for adoption of urine diversion in communities

Communities planning to implement urine diversion should identify locations where urine diversion systems are most appropriate and ensure such systems comply with existing regulations. Communities will need to gather data to estimate occupancy for different CI buildings by using existing occupancy surveys, existing metrics (e.g., water usage benchmarks), or novel surrogates for occupancy (e.g., wireless network activity (Depatla et al., 2015; Jagadeesh Simma et al., 2019; Mehmood et al., 2019; Saetta et al., 2021)) and estimate P recovery rates by applying per-occupant benchmarks (Table 1) to inform the locations for implementation of urine diversion. Once particular locations have been identified, building codes and zoning requirements may need to be altered to accommodate urine diversion systems as well as decentralized wastewater treatment. In addition to legal requirements, different industries have their own standards for construction and renovation of facilities. For example, the LEED certification process provides guidelines to contractors who specialize in green buildings (U.S. Green Building Council, 2023). By harmonizing a community's need for urine diversion systems with existing public and private standards, adoption may spread beyond a few model buildings or organizations.

Communities must also foster active collaboration with different stakeholders whose cooperation is necessary so that implementation of urine diversion systems leads to successful adoption. For example, the construction or renovation of facilities requires architects, engineers, contractors, plumbers, and other workers. Plumbers and their unions may have input on changes to plumbing codes, and once built, will oversee the operation and maintenance of these systems. Importantly, building occupants must be educated on urine diversion, such as on use of urine-diverting toilets (Abeysuriya et al., 2013; Blume and Winker, 2011; Domènech and Saurí, 2010). All these different stakeholders are embedded in organizations, such as hospitals, airports, and office buildings. Each organization has its own mission, priorities, financial circumstances, and staffing levels that must be considered. This is best accomplished through a program of coordinated stakeholder engagement that emphasizes benefits and wider values, which might be shared across these constituencies, such as cost savings or sustainability.

## 4 Conclusions

• The benefits of urine diversion in CI buildings are strongly influenced by occupancy count and occupancy time. These combined factors result in suitable CI building types to include hospitals, schools, office buildings, and airports.
• Buildings can accrue economic benefits from urine-recovered P and potable water savings that reduce the payback period of capital infrastructure costs of implementing urine diversion to <5 years. Potable water savings outweigh urine-recovered P for providing economic benefits by >50 × and provide an immediate incentive for implementing urine diversion systems.
• Urine diversion systems can be implemented at varying levels of decentralization based on community layout, organizational structure, socioeconomic and policy-relevant contexts. Per-occupant or per-building benchmarks for urine generation and P recovery can be applied to specific communities as a starting point to determine locations for urine diversion.

## 5 Methods

### 5.1 Building occupancy analysis

Different CI buildings were selected for analysis based on their expected occupancies, expected time spent in buildings, availability of benchmarking data in literature (Carvalho et al., 2013; Dziegielewski et al., 2000; Özlem Vurmaz and Boyacioglu, 2018), and if they have dedicated staff for managing building wastewater systems. Buildings were assigned a number from 1 to 3 in each category based on their performance in the category's criteria, with a 3 being well-performing. Numbers were summed for each building, and buildings with a final sum of greater than or equal to 8 (i.e., an average-performing building) were selected for additional analysis. A summary of CI building screening is found in Table S1. Low, average, and high occupancy scenarios were then calculated for selected CI buildings based on literature (Phillip et al., 1984; Bond et al., 1999; Liu et al., 2022; Hung et al., 2010; Williams, n.d.; OECD, n.d.; NCES, n.d.; Anthony, 2020; College Board, n.d.; Wood, n.d.; NCES, 1999; Azar and Menassa, 2012; Larson, 2021; United States Securities and Exchange Commission, 2008b, 2008a) and assumed values. Detailed calculations are found in Tables S2 through S13 for each selected CI building. Building occupants were separated into groups based on their expected time in a CI building, and occupancy was estimated based on specific groups that a specific CI building may be targeted for (e.g., guests in hotels, students in universities, passengers in airports). Conversion factors for targeted-occupants-to-other-occupants (e.g., staff) were used to determine occupancies of each group.

### 5.2 Urine and P generation rates in buildings

Urine generation rates were calculated based on previous analysis in literature (Landry and Boyer, 2016), using urine void volumes, urination frequencies, and expected hours awake in buildings to determine urination events and urine volumes per person per day. The following equations show the calculations used for the analysis. A single-point estimate of daily void volumes were assumed to be 1.65 L/d (male) and 1.62 L/d (female) from previous literature (Landry and Boyer, 2016). Total daytime voids were assumed to be 7 (male) and 8 (female) from previous literature (Landry and Boyer, 2016). Mean void volumes were then calculated for both males and females:

(1) $$\frac{Urine\;volume}{void}=\frac{\frac{Urine\;void\;volume}{d}}{\frac{voids}{d}}$$

Calculated mean voided volumes were within 10 % of estimates from a clinical study (median of 220 mL per void for males) (Huang Foen Chung and van Mastrigt, 2009). Average daily hours awake were assumed to be 17.6 h for both males and females from previous literature (Landry and Boyer, 2016). Urination frequencies were calculated to be 0.40 voids/h (male) and 0.45 voids/h (female) from previous literature (Landry and Boyer, 2016) by using the following equation:

(2) $$Urination\;frequency,\frac{voids}{h}=\frac{Total\;daytime\;voids,\;\frac{voids}{d}}{Average\;daily\;hours\;awake,\;\frac{h}{d}}$$

Urination events and urine volume produced were then calculated, assuming buildings have an equal percentage of males and females (i.e.,%male=%female=0.5).

(3) $$Urination\;events=\left(Building\;occupancy\right)\left(Hours\;occupied\right)\left({\left(\frac{voids}{h}\right)}_{male}\left(\%\;male\right)+{\left(\frac{voids}{h}\right)}_{female}\left(\%\;female\right)\right)\;$$

(4) $$Volume\;urine\;produced=\left(Urination\;events\right)\left({\left(\frac{urine\;volume}{void}\right)}_{male}\left(\%\;male\right)+{\left(\frac{urine\;volume}{void}\right)}_{female}\left(\%\;female\right)\right)\;$$

For buildings with 24-h occupants, first-morning urination events were modeled to have a higher P concentration and a lower void volume, based on literature values of urine osmolality and urine production (Bottin et al., 2016; Perrier et al., 2013; Suh et al., 2020). First-morning urine osmolality and production were seen to be ∼151 % and ∼75 % of that of afternoon samples, respectively (Perrier et al., 2013); this was calculated by adjusting only the first urination event for 24-h occupants:

(5) $$Volume\;urine\;produced=\left(Urination\;events-1\right)\left({\left(\frac{urine\;volume}{void}\right)}_{male}\left(\%\;male\right)+{\left(\frac{urine\;volume}{void}\right)}_{female}\left(\%\;female\right)\right)+\;First\;morning\;urine\;volume$$

(6) $$First\;morning\;urine\;volume=\left({\left(\frac{urine\;volume}{void}\right)}_{male}\left(\%\;male\right)+{\left(\frac{urine\;volume}{void}\right)}_{female}\left(\%\;female\right)\right)\left(0.75\right)$$

Total urine volumes in CI buildings were computed based on occupancy scenarios. An expected P concentration in urine was then applied to determine mass of P produced in CI buildings (Jagtap and Boyer, 2018; Rose et al., 2015). For buildings with 24-h occupants, the first morning urine volume was multiplied by an adjusted P concentration, i.e., the expected P concentration multiplied by the 151 % concentration factor, to determine the mass of P produced from first-morning urinations; this was then added to the mass of P produced from other urination events in the building (calculated with the expected P concentration). Adjusting for hours within buildings, the calculated mass of P produced per person per day were within 5 % of literature estimates for daily P excretion (approximately 700 mg P per day) (Prasad and Bhadauria, 2013). Detailed calculations can be found in Tables S14 and S15.

A sensitivity analysis was conducted on daily urine generation rates, void volumes, and urinary P concentrations to see their effects on P recovery rates. Monte Carlo simulations were run (*n* = 1000) with varied daily urine volumes (i.e., $\frac{Urine\;void\;volume}{d}$) and urine void volumes (i.e., $\frac{urine\;volume}{void}$), noting that total daytime voids no longer is a constant model input but is instead calculated. Gamma distributions for male and female daily urine volumes and urine void volumes were modeled from previous literature. Importantly, previous literature did not model males and females separately, so offsets used in the model were adjusted based on the discrepancy of the single-point estimates (e.g., single-point estimates for male daily urine volumes (1.65 L/d) were greater than that of females (1.62 L/d) by 0.03 L/d, so gamma distribution offsets were lower for females by 0.03 L/d (Rauch et al., 2003)). Urinary P concentrations were assumed to be normal, with a mean of 471.1 mg/L P and a standard deviation of 125 mg/L P, based on previous literature estimates of the range of urinary P concentrations (Meinzinger and Oldenburg, 2009; Rose et al., 2015). Based on Monte Carlo simulations of daily urine volumes, urine void volumes, and urinary P concentrations, ranges of potential P mass production from different CI buildings were created using the same calculations as in Tables S14 and S15.

### 5.3 Water benchmarking and water use savings in buildings

Water use benchmarks for different CI buildings were taken from previous literature (Carvalho et al., 2013; Dziegielewski et al., 2000; Özlem Vurmaz and Boyacioglu, 2018). A summary of benchmarks from previous literature can be found in Table S1. Water benchmarks were used to calculate the overall water usage of selected CI buildings in different occupancy scenarios. Urination flush volumes were then calculated based on assumptions of one flush for each urination event. Flush volumes were assumed to be the worst-case scenario based on national standards, i.e., 1.6 gallons per flush for toilets and 1.0 gallons per flush for urinals (U.S. Federal Register, 2022). Overall flush volumes were calculated for different occupancy scenarios, and water use savings were calculated, assuming no flush water used for a urine diversion scenario. Detailed calculations can be found in Tables S16 through S18.

### 5.4 Economic analysis of urine diversion in buildings

Costs of retrofitting buildings with urine diversion appliances and piping were estimated based on market averages and personal communications with a urine-diverting toilet company (Alibaba, 2022; J. D. Live, personal communication, September 27, 2022; Sloan, n.d.), assuming fifty occupants per wastewater appliance. Pipe requirements were assumed to be five meters of horizontal four-inch PVC pipe per appliance and five meters of vertical four-inch PVC pipe per building floor (Grundfos Product Center, n.d.). Building floors were assumed for each CI building type and occupancy scenario, as seen in Table S19. Profits from recovered nutrients were estimated based on single-point average (USDA ERS, 2021) and extreme (Quinn, 2021, 2023) P fertilizer costs. Profits from water savings were estimated based on assumed single-point average commercial water costs of $0.0015 per gal and extreme water costs (Tiseo, 2023). A summary of estimated costs and profits can be found in Tables 2, S20 and S21.

### 5.5 Framework for implementing urine diversion in communities

Existing zoning maps for a community can be accessed that show CI zoning for hospitals, schools, office buildings, and airports. Zoning maps can be cross-referenced with property data and city maps to identify CI buildings of interest where urine diversion can be implemented. Municipalities often publish Geographic Information System (GIS) layers that include zoning, and such layers can be used within GIS to color-code different CI buildings. Zoning blocks can then be screened using the decision tree detailed in Fig. 3, based on key parameters detailed in Table S22. Zoning blocks first should be screened for targeted CI buildings; if these buildings exist within the block, select “high-flow” buildings should be identified based on occupancy data (in general, “high-flow” corresponds with hospitals, airports, and schools). CI buildings surrounding “high-flow” buildings should be identified as “high-flow” or “low-flow”, based on occupancy data. Isolated “high-flow” buildings or multiple “high-flow” buildings nearby each other justify individual decentralized organization-specific facilities; “high-flow” buildings surrounded by “low-flow” buildings justify great block facilities that serve multiple CI buildings. If no “high-flow” buildings can be identified but there is a high density of “low-flow” CI buildings (e.g., office buildings), a semi-centralized supply and treatment facility may be justified. Otherwise, the zoning block is not viable for urine diversion. Additional characterization of the zoning block can be done to determine if different levels of decentralized urine treatment are feasible, using technical and economic analyses described in the above sections. Importantly, while this example framework is qualitative, the analysis done could also include quantitative analysis if community-scale data is available.

## Supplementary Materials

Supplementary material associated with this article can be found in the online version.

## CRediT authorship contribution statement

**Lucas Crane:** Writing – original draft, Investigation, Formal analysis, Conceptualization. **Ashton Merck:** Writing – review & editing, Conceptualization. **Shwetha Delanthamajalu:** Writing – review & editing, Conceptualization. **Khara Grieger:** Writing – review & editing, Funding acquisition, Conceptualization. **Anna-Maria Marshall:** Writing – review & editing, Funding acquisition, Conceptualization. **Treavor H. Boyer:** Writing – original draft, Supervision, Project administration, Funding acquisition, Conceptualization.

## Declaration of competing interest

The authors declare that they have no known competing financial interests or personal relationships that could have appeared to influence the work reported in this paper. Treavor Boyer is an editor at Water Research X.

## Data Availability

Data will be made available on request.

## Appendix Supplementary materials

Image, application 1
