# Supplementary material for: Benchmarks for urine volume generation and phosphorus mass recovery in commercial and institutional buildings
Source: Water Res X. 2024 May 8;23:100227. doi: 10.1016/j.wroa.2024.100227 (PMC11101975; doi:10.1016/j.wroa.2024.100227)
Supplement: Supplementary file 1 [file mmc1.docx]

**Benchmarks for urine volume generation and phosphorus mass recovery in commercial and institutional buildings**

**Lucas Crane^a,d^, Ashton Merck^b,d^, Shwetha Delanthamajalu^c,d^, Khara Grieger^b,d^, Anna-Maria Marshall^c,d^, Treavor H. Boyer^a,d*^**

**^a^ School of Sustainable Engineering and the Built Environment (SSEBE)**

**Arizona State University, PO Box 873005, Tempe, AZ 85287-3005, USA**

**^b^ Department of Applied Ecology**

**North Carolina State University, Raleigh NC 27606, USA**

**^c^ Department of Sociology**

**University of Illinois Urbana-Champaign**

**^d^ NSF Science and Technologies for Phosphorus Sustainability (STEPS) Center**

***Corresponding author.**

**Tel.: 1-480-965-7447**

**E-mail address: thboyer@asu.edu (T.H. Boyer).**

**SUPPLEMENTARY MATERIALS**

14 pages

22 tables

4 figures

**1. Supplementary Tables**

Table S1. Decision-making matrix for CI building selection. A score of “3” is well-performing, while a score of “1” is poor-performing.

| CI Building Type | High occupancy expected | High amount of time in building expected | Water benchmarking data available | Dedicated building manager/  janitorial staff | **Decision score** |
| --- | --- | --- | --- | --- | --- |
| Schools and colleges | 3 | 3 | 3 | 3 | **12** |
| Hotels and motels | 3 | 2 | 2 | 3 | **10** |
| Laundries/laundromats | 1 | 1 | 1 | 1 | **4** |
| Office buildings | 2 | 3 | 3 | 3 | **11** |
| Hospital/medical office | 3 | 3 | 3 | 3 | **12** |
| Restaurants | 2 | 1 | 3 | 2 | **8** |
| Food stores | 2 | 2 | 1 | 1 | **6** |
| Auto shops | 1 | 2 | 1 | 1 | **5** |
| Car washes | 1 | 1 | 1 | 1 | **4** |
| Airports | 3 | 1 | 2 | 3 | **9** |

Table S2. Occupancy calculations for hospitals.

| Occupancy scenario | Number of beds | Average hospital occupancy percentage (Phillip et al., 1984) | Patient occupancy | Average employees per occupied hospital bed (Bond et al., 1999) | Employee occupancy |
| --- | --- | --- | --- | --- | --- |
| Low | 15 | 0.462 | 6.93 | 5.28 | 36.59 |
| Medium | 249.5 | 0.778 | 194.111 | 5.28 | 1024.91 |
| High | 500 | 0.825 | 412.5 | 5.28 | 2178.01 |

Table S3. Occupancy calculations for hotels.

| Occupancy scenario | Number of rooms (Liu et al., 2022) | Occupancy rate (average) (Liu et al., 2022) | Average guests per room | Guest occupancy | Average staff per room (Hung et al., 2010) | Staff occupancy |
| --- | --- | --- | --- | --- | --- | --- |
| Low (minimum) | 78 | 0.6922 | 2 | 53.99 | 0.30 | 16.21 |
| Medium (average) | 271.03 | 0.6922 | 2 | 187.61 | 0.30 | 56.34 |
| High (maximum) | 2882 | 0.6922 | 2 | 1994.92 | 0.30 | 599.05 |

Table S4. Occupancy calculations for elementary schools.

| Occupancy scenario | Student occupancy | Average students per teacher (*Teachers - Students per Teaching Staff - OECD Data*, n.d.) | Teachers | Percent of non-teaching staff in total (*Teacher Characteristics and Trends*, n.d.) | Non-teaching staff occupancy |
| --- | --- | --- | --- | --- | --- |
| Low | 50 | 15.3 | 3.27 | 0.34 | 1.69 |
| Medium (average) | 424 (Williams, n.d.) | 15.3 | 27.71 | 0.34 | 14.28 |
| High | 2000 | 15.3 | 130.72 | 0.34 | 67.34 |

Table S5. Occupancy calculations for secondary schools.

| Occupancy scenario | Student occupancy | Average students per teacher (*Teachers - Students per Teaching Staff - OECD Data*, n.d.) | Teachers | Percent of non-teaching staff in total (*Teacher Characteristics and Trends*, n.d.) | Non-teaching staff occupancy |
| --- | --- | --- | --- | --- | --- |
| Low | 50 | 15.3 | 3.27 | 0.34 | 1.68 |
| Medium (average) | 696 (Williams, n.d.) | 15.3 | 45.49 | 0.34 | 23.43 |
| High | 4000 | 15.3 | 261.44 | 0.34 | 134.68 |

Table S6. Occupancy calculations for universities/colleges.

| Occupancy scenario | Student occupancy | Percent of students living on-campus (Anthony, 2020) | Off-campus student occupancy | On-campus student occupancy | Average students per  Faculty (*Digest of Education Statistics, 1999*, n.d.) | Faculty occupancy | Average students per non-faculty staff (*Digest of Education Statistics, 1999*, n.d.) | Non-faculty staff occupancy |
| --- | --- | --- | --- | --- | --- | --- | --- | --- |
| Low | 500 | 0.22 | 390 | 110 | 14 | 35.71 | 10 | 50 |
| Medium (average) | 6354 (*Understand College Campus and Student Body Size – BigFuture \| College Board*, n.d.; Wood, n.d.) | 0.22 | 4956.12 | 1397.88 | 14 | 453.86 | 10 | 653.4 |
| High | 70000 | 0.22 | 54600 | 15400 | 14 | 5000 | 10 | 7000 |

Table S7. Occupancy calculations for office buildings.

| Occupancy scenario | Occupancy (Azar & Menassa, 2012) | Number of floors | Building size (sf) |
| --- | --- | --- | --- |
| Low (minimum) | 6 | 1 | 2391 |
| Medium (average) | 127.6 | 3.2 | 54486.6 |
| High (maximum) | 258 | 7 | 152113 |

Table S8. Occupancy calculations for restaurants, slow hours.

| Occupancy scenario | Number of tables | Guests per table | Guest length of stay (h) | Slow hour occupancy percentage | Number of slow hours | Guests per slow hour | Guests during slow hours |
| --- | --- | --- | --- | --- | --- | --- | --- |
| Low | 10 | 3 | 1 | 0.2 | 2 | 6 | 12 |
| Medium | 25 | 3 | 1 | 0.2 | 2 | 15 | 30 |
| High | 50 | 3 | 1 | 0.2 | 2 | 30 | 60 |

Table S9. Occupancy calculations for restaurants, normal hours.

| Occupancy scenario | Number of tables | Guests per table | Guest length of stay (h) | Normal hour occupancy percentage | Number of normal hours | Guests per normal hour | Guests during normal hours |
| --- | --- | --- | --- | --- | --- | --- | --- |
| Low | 10 | 3 | 1 | 0.6 | 8 | 18 | 144 |
| Medium | 25 | 3 | 1 | 0.6 | 8 | 45 | 360 |
| High | 50 | 3 | 1 | 0.6 | 8 | 90 | 720 |

Table S10. Occupancy calculations for restaurants, peak hours.

| Occupancy scenario | Number of tables | Guests per table | Guest length of stay (h) | Peak hour occupancy percentage | Number of peak hours | Guests per peak hour | Guests during peak hours |
| --- | --- | --- | --- | --- | --- | --- | --- |
| Low | 10 | 3 | 1 | 1.0 | 2 | 30 | 60 |
| Medium | 25 | 3 | 1 | 1.0 | 2 | 75 | 150 |
| High | 50 | 3 | 1 | 1.0 | 2 | 150 | 300 |

Table S11. Occupancy calculations for restaurants, total guests.

| Occupancy scenario | Number of tables | Guests per table | Guest capacity | Total daily guest occupancy |
| --- | --- | --- | --- | --- |
| Low | 10 | 3 | 30 | 216 |
| Medium | 25 | 3 | 75 | 540 |
| High | 50 | 3 | 150 | 1080 |

Table S12. Occupancy calculations for restaurants, employees.

| Occupancy scenario | Back of house employees per guest capacity (Larson, 2021) | Back of house employees | Front of house employees per table (Larson, 2021) | Front of house employees | Managers (Larson, 2021) | Employee occupancy per shift | Total daily employees (assume 3 shifts) |
| --- | --- | --- | --- | --- | --- | --- | --- |
| Low | 0.1 | 3 | 0.25 | 2.5 | 1 | 6.5 | 19.5 |
| Medium | 0.1 | 7.5 | 0.25 | 6.25 | 1 | 14.75 | 44.25 |
| High | 0.1 | 15 | 0.25 | 12.5 | 1 | 28.5 | 85.5 |

Table S13. Occupancy calculations for airports.

| Occupancy scenario | Number of yearly passengers | Daily passenger occupancy | Passengers per employee (*Annual Report Pursuant to Section 13 or 15(d) of the Securities Exchange Act of 1934 \| American Airlines, Inc.*, 2008; *Annual Report Pursuant to Section 13 or 15(d) of the Securities Exchange Act of 1934 \| Southwest Airlines Co.*, 2008) | Employees | Percent employees in flight (*Annual Report Pursuant to Section 13 or 15(d) of the Securities Exchange Act of 1934 \| Southwest Airlines Co.*, 2008) | Flight staff occupancy | Airport staff occupancy |
| --- | --- | --- | --- | --- | --- | --- | --- |
| Low | 100000 | 273.97 | 2000 | 50 | 0.436 | 21.81 | 28.19 |
| Medium | 4000000 | 10958.90 | 2000 | 2000 | 0.436 | 872.31 | 1127.69 |
| High | 20000000 | 54794.52 | 2000 | 10000 | 0.436 | 4361.53 | 5638.47 |

Table S14. Detailed calculations for phosphorus generation.

| Building type | Time awake group 1 (h) | Time awake group 2 (h) | Time awake group 3 (h) | Urine void volume, male (L/d) | Total Daytime Voids, male | Mean voided volume, male (L/void) | Average hours awake, male (h) | Urination frequency, male (void/h) | Urine void volume, female (L/d) | Total Daytime Voids, female | Mean voided volume, female (L/void) | Average hours awake, female (h) | Urination frequency, female (void/h) | Percent female |
| --- | --- | --- | --- | --- | --- | --- | --- | --- | --- | --- | --- | --- | --- | --- |
| Hospitals | 17.6 | 12 |  | 1.65 | 7 | 0.24 | 17.60 | 0.40 | 1.62 | 8.00 | 0.20 | 17.60 | 0.45 | 0.50 |
| Hotels and motels | 4 | 8 |  | 1.65 | 7 | 0.24 | 17.60 | 0.40 | 1.62 | 8.00 | 0.20 | 17.60 | 0.45 | 0.50 |
| Office buildings | 8 |  |  | 1.65 | 7 | 0.24 | 17.60 | 0.40 | 1.62 | 8.00 | 0.20 | 17.60 | 0.45 | 0.50 |
| Restaurants | 1 | 4.5 |  | 1.65 | 7 | 0.24 | 17.60 | 0.40 | 1.62 | 8.00 | 0.20 | 17.60 | 0.45 | 0.50 |
| Elementary schools | 8 |  |  | 1.65 | 7 | 0.24 | 17.60 | 0.40 | 1.62 | 8.00 | 0.20 | 17.60 | 0.45 | 0.50 |
| Secondary schools | 8 |  |  | 1.65 | 7 | 0.24 | 17.60 | 0.40 | 1.62 | 8.00 | 0.20 | 17.60 | 0.45 | 0.50 |
| Universities  /colleges | 3 | 8 | 17.6 | 1.65 | 7 | 0.24 | 17.60 | 0.40 | 1.62 | 8.00 | 0.20 | 17.60 | 0.45 | 0.50 |
| Airports | 1.5 | 1 | 8 | 1.65 | 7 | 0.24 | 17.60 | 0.40 | 1.62 | 8.00 | 0.20 | 17.60 | 0.45 | 0.50 |

Table S15. Detailed calculations for phosphorus generation, continued.

| Building type | Volume of urine produced per person per day (L/cap-d), group 1 | Volume of urine produced per person per day (L/cap-d), group 2 | Volume of urine produced per person per day (L/cap-d), group 3 | Total volume of urine produced per day, low scenario (L/day) | Total volume of urine produced per day, medium scenario (L/day) | Total volume of urine produced per day, high scenario (L/day) | Average phosphorus concentration in urine (kg/L) | P concentration factor for first-morning urination event for 24-h occupant | Mass P per person per day (g/cap-d), group 1 | Mass P per person per day (g/cap-day), group 2 | Mass P per person per day (g/cap-day), group 3 | Mass P per day (kg/d), low scenario | Mass P per day (kg/d), medium scenario | Mass P per day (kg/d), high scenario |
| --- | --- | --- | --- | --- | --- | --- | --- | --- | --- | --- | --- | --- | --- | --- |
| Hospitals | 1.57 | 1.12 | 0.00 | 51.87 | 1452.83 | 3087.37 | 4.11E-04 | 1.51 | 0.68 | 0.46 | 0.00 | 2.15E-02 | 0.60 | 1.28 |
| Hotels and motels | 0.37 | 0.75 | 0.00 | 32.28 | 112.15 | 1192.52 | 4.11E-04 |  | 0.15 | 0.31 | 0.00 | 1.33E-02 | 0.05 | 0.49 |
| Office buildings | 0.75 | 0.00 | 0.00 | 4.48 | 95.31 | 267.41 | 4.11E-04 |  | 0.31 | 0.00 | 0.00 | 1.84E-03 | 0.04 | 0.11 |
| Restaurants | 0.09 | 0.42 | 0.00 | 28.36 | 69.01 | 136.76 | 4.11E-04 |  | 0.04 | 0.17 | 0.00 | 1.17E-02 | 0.03 | 0.06 |
| Elementary schools | 0.75 | 0.00 | 0.00 | 41.05 | 348.07 | 1641.85 | 4.11E-04 |  | 0.31 | 0.00 | 0.00 | 1.69E-02 | 0.14 | 0.67 |
| Secondary schools | 0.75 | 0.00 | 0.00 | 41.05 | 571.37 | 3283.71 | 4.11E-04 |  | 0.31 | 0.00 | 0.00 | 1.69E-02 | 0.23 | 1.35 |
| Universities/  colleges | 0.28 | 0.75 | 1.57 | 345.81 | 4394.61 | 48413.96 | 4.11E-04 | 1.51 | 0.12 | 0.31 | 0.68 | 1.46E-01 | 1.85 | 20.36 |
| Airports | 0.14 | 0.09 | 0.75 | 61.47 | 2458.63 | 12293.13 | 4.11E-04 |  | 0.06 | 0.04 | 0.31 | 2.53E-02 | 1.01 | 5.05 |

Table S16. Detailed calculations for water savings.

| Building type | 2000 toilet flush volume (gal per flush) | 2000 urinal flush volume (gal per flush) | Percent of male urinations in urinal | Percent male in buildings | Male urination events, group 1 | Male urination volume, group 1 | Female urination events, group 1 | Female urination volume, group 1 | Flush volume, toilet, group 1 (gal) | Flush volume, urinal, group 1 (gal) | Total flush volume per person, group 1 (gal) |
| --- | --- | --- | --- | --- | --- | --- | --- | --- | --- | --- | --- |
| Hospitals | 1.6 | 1 | 0.95 | 0.50 | 3.5 | 0.74 | 4 | 0.74 | 6.68 | 3.33 | 10.01 |
| Hotels and motels | 1.6 | 1 | 0.95 | 0.50 | 0.80 | 0.19 | 0.91 | 0.18 | 1.51 | 0.76 | 2.27 |
| Office buildings | 1.6 | 1 | 0.95 | 0.50 | 1.59 | 0.38 | 1.82 | 0.37 | 3.03 | 1.51 | 4.55 |
| Restaurants | 1.6 | 1 | 0.95 | 0.50 | 0.20 | 0.05 | 0.23 | 0.05 | 0.38 | 0.19 | 0.57 |
| Elementary schools | 1.6 | 1 | 0.95 | 0.50 | 1.59 | 0.38 | 1.82 | 0.37 | 3.04 | 1.51 | 4.55 |
| Secondary schools | 1.6 | 1 | 0.95 | 0.50 | 1.59 | 0.38 | 1.82 | 0.37 | 3.04 | 1.51 | 4.55 |
| Universities/colleges | 1.6 | 1 | 0.95 | 0.50 | 0.60 | 0.14 | 0.68 | 0.14 | 1.14 | 0.57 | 1.71 |
| Airports | 1.6 | 1 | 0.95 | 0.50 | 0.30 | 0.07 | 0.34 | 0.07 | 0.57 | 0.28 | 0.85 |

Table S17. Detailed calculations for water savings, continued.

| Building type | Male urination events, group 2 | Male urination volume, group 2 | Female urination events, group 2 | Female urination volume, group 2 | Flush volume, toilet, group 2 (gal) | Flush volume, urinal, group 2 (gal) | Total flush volume per person, group 2 (gal) | Male urination events, group 3 | Male urination volume, group 3 | Female urination events, group 3 | Female urination volume, group 3 | Flush volume, toilet, group 3 (gal) | Flush volume, urinal, group 3 (gal) | Total flush volume per person, group 3 (gal) | 2000 Total flush volume, low scenario (gal) | 2000 Total flush volume, medium scenario (gal) | 2000 Total flush volume, high scenario (gal) |
| --- | --- | --- | --- | --- | --- | --- | --- | --- | --- | --- | --- | --- | --- | --- | --- | --- | --- |
| Hospitals | 2.39 | 0.56 | 2.73 | 0.55 | 4.55 | 2.27 | 6.82 | 0.00 | 0.00 | 0.00 | 0.00 | 0.00 | 0.00 | 0.00 | 318.94 | 8934 | 18985 |
| Hotels and motels | 1.59 | 0.38 | 1.82 | 0.37 | 3.04 | 1.51 | 4.55 | 0.00 | 0.00 | 0.00 | 0.00 | 0.00 | 0.00 | 0.00 | 196.50 | 682.79 | 7260 |
| Office buildings | 0.00 | 0.00 | 0.00 | 0.00 | 0.00 | 0.00 | 0.00 | 0.00 | 0.00 | 0.00 | 0.00 | 0.00 | 0.00 | 0.00 | 27.29 | 580.29 | 1628 |
| Restaurants | 0.89 | 0.21 | 1.02 | 0.21 | 1.71 | 0.85 | 2.56 | 0.00 | 0.00 | 0.00 | 0.00 | 0.00 | 0.00 | 0.00 | 172.67 | 420.17 | 832.66 |
| Elementary schools | 0.00 | 0.00 | 0.00 | 0.00 | 0.00 | 0.00 | 0.00 | 0.00 | 0.00 | 0.00 | 0.00 | 0.00 | 0.00 | 0.00 | 249.90 | 2119 | 9996 |
| Secondary schools | 0.00 | 0.00 | 0.00 | 0.00 | 0.00 | 0.00 | 0.00 | 0.00 | 0.00 | 0.00 | 0.00 | 0.00 | 0.00 | 0.00 | 249.90 | 3479 | 19992 |
| Universities/  colleges | 1.59 | 0.38 | 1.82 | 0.37 | 3.04 | 1.51 | 4.55 | 3.5 | 0.74 | 4 | 0.74 | 6.68 | 3.325 | 10.01 | 2155 | 27392 | 301764 |
| Airports | 0.20 | 0.05 | 0.23 | 0.05 | 0.38 | 0.19 | 0.57 | 1.59 | 0.38 | 1.82 | 0.37 | 3.04 | 1.51 | 4.55 | 374.22 | 14969 | 74845 |

Table S18. Detailed calculations for water savings, continued.

| Building type | Percent of total population for group 1, low scenario | Percent of total population for group 1, medium scenario | Percent of total population for group 1, high scenario | Total population, low scenario | Total population, medium scenario | Total population, high scenario | Water use benchmark for indoor | Units | Percent indoor use of total use | Total Water Use, low (gal/day) | Total Water Use, medium (gal/day) | Total Water Use, high (gal/day) | Percent water savings from 2000, low | Percent water savings from 2000, medium | Percent water savings from 2000, high |
| --- | --- | --- | --- | --- | --- | --- | --- | --- | --- | --- | --- | --- | --- | --- | --- |
| Hospitals | 0.16 | 0.16 | 0.16 | 43.52 | 1219.02 | 2590.51 | 315.00 (Healthcare Facilities Today, 2013) | gal/occupied bed/day | N/A | 2182.95 | 61144.97 | 129937.50 | 0.15 | 0.15 | 0.15 |
| Hotels and motels | 0.77 | 0.77 | 0.77 | 70.20 | 243.94 | 2593.97 | 127.40 (Dziegielewski et al., 2000) | gal/day/occupied room | N/A | 6878.38 | 23900.61 | 254147.39 | 0.03 | 0.03 | 0.03 |
| Office buildings | 1.00 | 1.00 | 1.00 | 6.00 | 127.60 | 358.00 | 12.50 (Dziegielewski et al., 2000) | gal/employee/day for indoor use | 0.39 | 190.63 | 4053.96 | 11373.96 | 0.14 | 0.14 | 0.14 |
| Restaurants | 0.92 | 0.92 | 0.93 | 235.50 | 584.25 | 1165.50 | 7.50 (Dziegielewski et al., 2000) | gal/meal served total | N/A | 1620.00 | 4050.00 | 8100.00 | 0.11 | 0.10 | 0.10 |
| Elementary schools | 1.00 | 1.00 | 1.00 | 54.95 | 465.99 | 2198.06 | 9.00 (Dziegielewski et al., 2000) | gal/school day/student for indoor use | 0.18 | 2493.75 | 21147.00 | 99750.00 | 0.10 | 0.10 | 0.10 |
| Secondary schools | 1.00 | 1.00 | 1.00 | 54.95 | 764.92 | 4396.12 | 9.00 (Dziegielewski et al., 2000) | gal/school day/student for indoor use | 0.18 | 2493.75 | 34713.00 | 199500.00 | 0.10 | 0.10 | 0.10 |
| Universities/  colleges | 0.82 | 0.82 | 0.82 | 585.71 | 7443.26 | 82000.00 | 9.00 (Dziegielewski et al., 2000) | gal/school day/student for indoor use | 0.18 | 24937.50 | 316905.75 | 3491250.00 | 0.09 | 0.09 | 0.09 |
| Airports | 0.93 | 0.93 | 0.93 | 323.97 | 12958.90 | 64794.52 | 22.66 (Özlem Vurmaz & Boyacioglu, 2018) | L potable/passenger | 1.00 | 1639.82 | 65592.82 | 327964.11 | 0.23 | 0.23 | 0.23 |

Table S19. Building story assumptions for cost analysis.

| Building type | Stories for low scenario | Stories for medium scenario | Stories for high scenario |
| --- | --- | --- | --- |
| Hospitals | 1 | 3 | 5 |
| Hotels and motels | 1 | 5 | 10 |
| Office buildings | 1 | 5 | 10 |
| Restaurants | 1 | 1 | 1 |
| Elementary schools | 1 | 1 | 2 |
| Secondary schools | 1 | 1 | 2 |
| Universities/colleges | 2 | 3 | 4 |
| Airports | 1 | 2 | 3 |

Table S20. Economic analysis of urine diversion systems in CI buildings at low and high occupancy scenarios, including phosphorus fertilizer profit (PF) ($/d), water savings (WS) ($/d), wastewater appliance costs (WWC) ($), and time to break even on costs (BE) (y).

| **Building type** | **Low scenario** | | | | **High scenario** | | | |
| --- | --- | --- | --- | --- | --- | --- | --- | --- |
|  | **PF** | **WS** | **WWC** | **BE** | **PF** | **WS** | **WWC** | **BE** |
| **Hospitals** | 0.09 | 0.48 | -510 | **2.4** | 5.3 | 28 | -26,000 | **2.1** |
| **Hotels** | 0.06 | 0.29 | -710 | **5.5** | 2.1 | 11 | -26,100 | **5.5** |
| **Office buildings** | 0.01 | 0.04 | -510 | **28** | 0.46 | 2.4 | -3,600 | **3.4** |
| **Restaurants** | 0.05 | 0.26 | -2,400 | **21** | 0.24 | 1.3 | -12,000 | **22** |
| **Elementary schools** | 0.07 | 0.38 | -560 | **3.4** | 2.8 | 15 | -22,000 | **3.4** |
| **Secondary schools** | 0.07 | 0.38 | -560 | **3.4** | 5.7 | 30 | -44,000 | **3.4** |
| **Universities/**  **colleges** | 0.61 | 3.2 | -5,900 | **4.2** | 85 | 450 | -820,000 | **4.2** |
| **Airports** | 0.11 | 0.56 | -3,300 | **13** | 21 | 110 | -650,000 | **13** |

Table S21. Range of times to break even (BE) based on extreme water prices (Tiseo, 2023) and phosphorus fertilizer costs for medium occupancy scenario (Quinn, 2021, 2023).

| **Building type** | **Water prices** | | | | **Phosphorus fertilizer costs** | | | |
| --- | --- | --- | --- | --- | --- | --- | --- | --- |
|  | **Low price ($/gal)** | **Low BE (y)** | **High price ($/gal)** | **High BE (y)** | **Low cost ($/kg P)** | **Low BE (y)** | **High cost ($/kg P)** | **High BE (y)** |
| **Hospitals** | 0.0002 | **8.7** | 0.025 | **0.15** | 1.9 | **2.3** | 4.7 | **2.1** |
| **Hotels** | 0.0002 | **23** | 0.025 | **0.39** | 1.9 | **6.1** | 4.7 | **5.4** |
| **Office buildings** | 0.0002 | **14** | 0.025 | **0.24** | 1.9 | **3.8** | 4.7 | **3.4** |
| **Restaurants** | 0.0002 | **88** | 0.025 | **1.5** | 1.9 | **24** | 4.7 | **21** |
| **Elementary schools** | 0.0002 | **14** | 0.025 | **0.24** | 1.9 | **3.7** | 4.7 | **3.3** |
| **Secondary schools** | 0.0002 | **14** | 0.025 | **0.24** | 1.9 | **3.7** | 4.7 | **3.3** |
| **Universities/**  **colleges** | 0.0002 | **17** | 0.025 | **0.29** | 1.9 | **4.6** | 4.7 | **4.1** |
| **Airports** | 0.0002 | **55** | 0.025 | **0.94** | 1.9 | **15** | 4.7 | **13** |

Table S22. Key parameters and tools in approach for community-wide implementation of urine diversion.

| **Parameter** | **Tool used for analysis** |
| --- | --- |
| “High-flow” CI building count | Zoning maps |
| “Low-flow” CI building count | Zoning maps |
| “High-flow” CI building density | Zoning maps |
| “Low-flow” CI building density | Zoning maps |
| Proximity of low-flow buildings to high-flow buildings | Zoning maps |
| CI building occupancy count | Building occupancy datasets, e.g., surveys or wireless network activity |
| CI building occupancy time | Building occupancy datasets, e.g., surveys or wireless network activity |

**2. Supplementary Figures**

**Figure S1**. Calculated phosphorus mass recovery rates for low and high occupancy scenarios for different CI building types.

**Figure S2.** Urine volume generation rates for different occupancy scenarios for different CI building types.


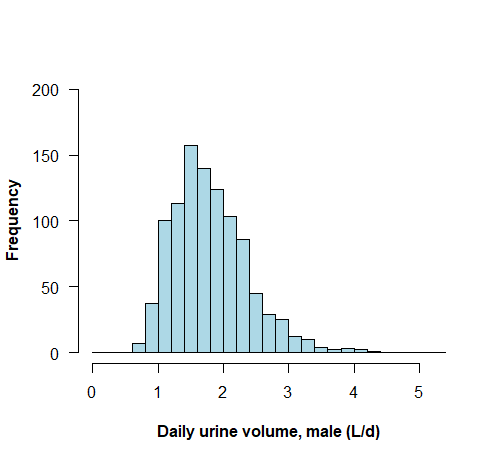

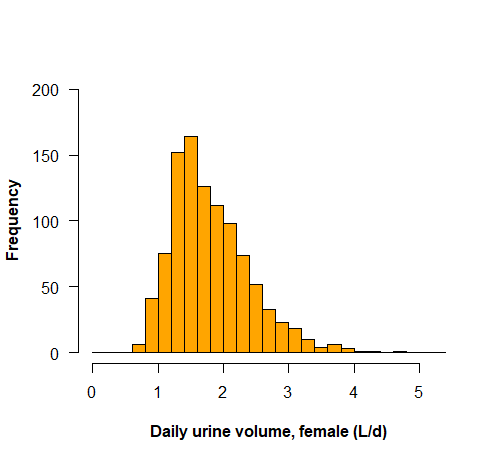


**(a)**

**(b)**


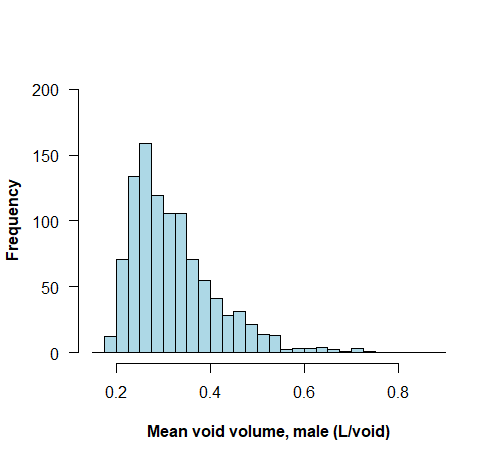

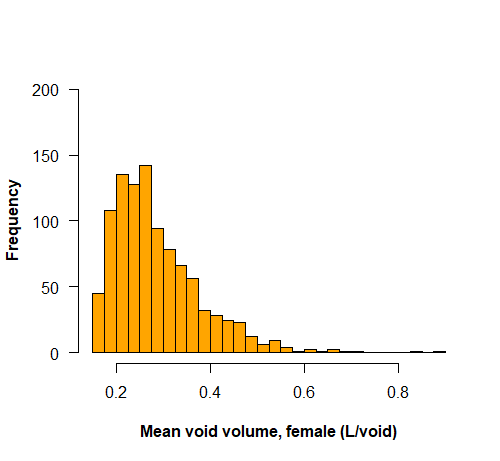


**(c)**

**(d)**

**Figure S3**. Range of daily urination volumes for (a) males and (b) females, and void volumes for (c) males and (d) females (Rauch et al., 2003).

**
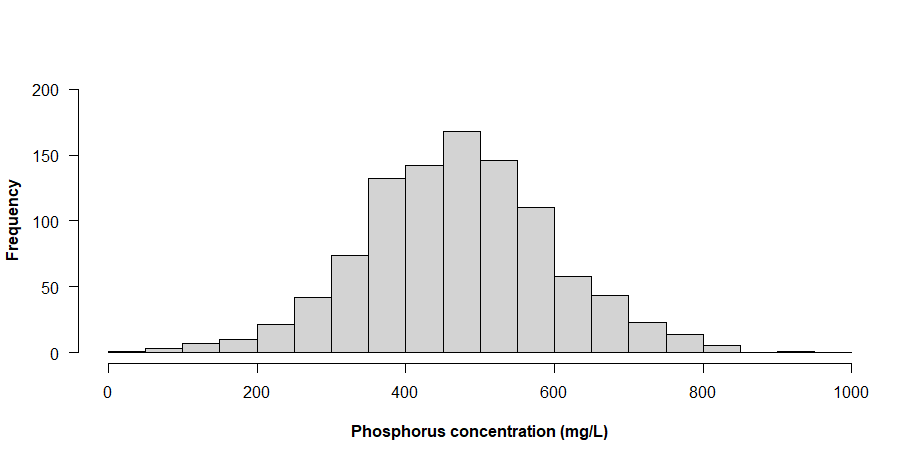
**

**Figure S4**. Range of urinary phosphorus concentrations (Meinzinger & Oldenburg, 2009; Rose et al., 2015).

**3. Supplementary References**

*Annual Report Pursuant to Section 13 or 15(d) of the Securities Exchange Act of 1934 | American Airlines, Inc.* (2008). United States Securities and Exchange Commission. https://www.sec.gov/Archives/edgar/data/4515/000000451509000008/aa120810k.htm

*Annual Report Pursuant to Section 13 or 15(d) of the Securities Exchange Act of 1934 | Southwest Airlines Co.* (2008). United States Securities and Exchange Commission. https://www.sec.gov/Archives/edgar/data/92380/000119312509015591/d10k.htm

Anthony, J. (2020, March 9). *47 Essential Student Housing Statistics You Must Learn: 2021/2022 Data & Demographics*. Financesonline.Com. https://financesonline.com/student-housing-statistics/

Azar, E., & Menassa, C. C. (2012). A comprehensive analysis of the impact of occupancy parameters in energy simulation of office buildings. *Energy and Buildings*, *55*, 841–853. https://doi.org/10.1016/j.enbuild.2012.10.002

Bond, C. A., Raehl, C. L., Pitterle, M. E., & Franke, T. (1999). Health Care Professional Staffing, Hospital Characteristics, and Hospital Mortality Rates. *Pharmacotherapy: The Journal of Human Pharmacology and Drug Therapy*, *19*(2), 130–138. https://doi.org/10.1592/phco.19.3.130.30915

*Digest of Education Statistics, 1999*. (n.d.). National Center for Education Statistics. Retrieved October 29, 2022, from https://nces.ed.gov/programs/digest/d99/d99t229.asp

Dziegielewski, B., Kiefer, J. C., Opitz, E. M., Porter, G. A., Lantz, G. L., DeOreo, W. B., Mayer, P. W., & Nelson, J. O. (2000). *Commercial and Institutional End Uses of Water*. AWWA Research Foundation. https://www.waterrf.org/resource/commercial-and-institutional-end-uses-water

Healthcare Facilities Today. (2013). *EPA: Hospitals use most water, but are not among top facilities tracking use*. Healthcare Facilities Today. https://www.healthcarefacilitiestoday.com/posts/EPA-Hospitals-use-most-water-but-are-not-among-top-facilities-tracking-use--284

Hung, W.-T., Shang, J.-K., & Wang, F.-C. (2010). Pricing determinants in the hotel industry: Quantile regression analysis. *International Journal of Hospitality Management*, *29*(3), 378–384. https://doi.org/10.1016/j.ijhm.2009.09.001

Larson, S. (2021, November 11). *How Many Employees Does it Take to Run a Restaurant?* Escoffier. https://www.escoffier.edu/blog/food-entrepreneurship/how-many-employees-does-it-take-to-run-a-restaurant/

Liu, P., Wu, L., & Li, X. (Robert). (2022). What can hotels learn from the last recovery? Examining hotel occupancy rate and the guest experience. *International Journal of Hospitality Management*, *103*, 103200. https://doi.org/10.1016/j.ijhm.2022.103200

Meinzinger, F., & Oldenburg, M. (2009). Characteristics of source-separated household wastewater flows: A statistical assessment. *Water Science and Technology: A Journal of the International Association on Water Pollution Research*, *59*(9), 1785–1791. https://doi.org/10.2166/wst.2009.185

Özlem Vurmaz, M., & Boyacioglu, H. (2018). Airport Water Consumption Footprinting. *Environment and Ecology Research*, *6*(6), 519–524. https://doi.org/10.13189/eer.2018.060601

Phillip, P. J., Mullner, R., & Andes, S. (1984). Toward a better understanding of hospital occupancy rates. *Health Care Financing Review*, *5*(4), 53–61.

Quinn, R. (2021, January 13). *Prices for Most Fertilizers Continue to Move Higher at Start of 2021*. DTN Progressive Farmer. https://www.dtnpf.com/agriculture/web/ag/news/crops/article/2021/01/13/prices-fertilizers-continue-move

Quinn, R. (2023, February 1). *Average UAN28 Fertilizer Price Drops 9% During January 2023*. DTN Progressive Farmer. https://www.dtnpf.com/agriculture/web/ag/news/crops/article/2023/02/01/average-uan28-price-drops-9-january

Rauch, W., Brockmann, D., Peters, I., Larsen, T. A., & Gujer, W. (2003). Combining urine separation with waste design: An analysis using a stochastic model for urine production. *Water Research*, *37*(3), 681–689. https://doi.org/10.1016/s0043-1354(02)00364-0

Rose, C., Parker, A., Jefferson, B., & Cartmell, E. (2015). The Characterization of Feces and Urine: A Review of the Literature to Inform Advanced Treatment Technology. *Critical Reviews in Environmental Science and Technology*, *45*(17), 1827–1879. https://doi.org/10.1080/10643389.2014.1000761

*Teacher characteristics and trends*. (n.d.). National Center for Education Statistics. Retrieved October 29, 2022, from https://nces.ed.gov/fastfacts/display.asp?id=28

*Teachers—Students per teaching staff—OECD Data*. (n.d.). TheOECD. Retrieved October 30, 2022, from http://data.oecd.org/teachers/students-per-teaching-staff.htm

Tiseo, I. (2023, February 6). *Lowest water prices in select cities globally 2021 | Statista*. Statista. https://www.statista.com/statistics/478888/leading-cities-based-on-lowest-freshwater-prices/

*Understand College Campus and Student Body Size – BigFuture | College Board*. (n.d.). Retrieved October 30, 2022, from https://bigfuture.collegeboard.org/plan-for-college/college-basics/types-of-colleges/understand-college-campus-student-body-size

USDA ERS. (2021). *Fertilizer Use and Price*. U.S. Department of Agriculture. https://www.ers.usda.gov/data-products/fertilizer-use-and-price.aspx

Williams, D. T. (n.d.). *THE DIMENSIONS OF EDUCATION: RECENT RESEARCH ON SCHOOL SIZE*. 23.

Wood, S. (n.d.). *10 Colleges With the Most Undergraduate Students*. U.S. News & World Report. Retrieved October 30, 2022, from https://www.usnews.com/education/best-colleges/the-short-list-college/articles/colleges-with-the-most-undergraduates
